# Evaluation of enteral and parenteral hyaluronic acid in induced ischemic skin flaps in rats: a double-blinded and randomized study

**DOI:** 10.1590/acb395924

**Published:** 2024-09-09

**Authors:** Marina Frazatti Gallina, Ivan Felismino Charas dos Santos, Bruna Martins da Silva, Guilherme Cirino Coelho Pereira, Lucas Fernando Sérgio Gushiken, Claudia Helena Pellizzon, Miriam Harumi Tsunemi, Sandro de Vargas Schons, Fernando do Carmo Silva, Kamile Daguano Sena, Vinicius dos Santos Rosa

**Affiliations:** 1 Universidade Estadual Paulista – School of Veterinary Medicine and Animal Science – Postgraduate Program in Animal Biotechnology – Botucatu (SP) –Brazil.; 2 Universidade Federal de Rondônia – Department of Veterinary Medicine – Rolim de Moura (RO) – Brazil.; 3 Universidade Cruzeiro do Sul – Department of Veterinary Medicine – São Paulo (SP) – Brazil.; 4 Universidade Estadual Paulista – School of Veterinary Medicine and Animal Science – Botucatu (SP) – Brazil.; 5 Universidade Estadual de Campinas – Hematology and Transfusion Center – Campinas (SP) – Brazil.; 6 Universidade Estadual Paulista – Institute of Biosciences – Department of Structural and Functional Biology – Botucatu (SP) – Brazil.; 7 Universidade Estadual Paulista – Institute of Biosciences – Department of Biostatistics – Botucatu (SP) – Brazil.; 8 Universidade do Oeste Paulista – Department of Veterinary Medicine – Presidente Prudente (SP) – Brazil.

**Keywords:** Wound Healing, Surgery, Plastic, Inflammatory Cytocines, Hyaluronic Acid

## Abstract

**Purpose:**

To evaluate exogenous hyaluronic acid (HA) derived from bacterial fermentation through enteral and parenteral routes in ischemic skin flaps induced in rats, using clinical and histological exams; and interleukins (IL) as tissue inflammatory biomarkers.

**Methods:**

Sixty-four male adults Wistar rats with ischemic skin flaps on the dorsum were randomized into four groups, based on the treatment protocol: subcutaneous administration of saline solution (0.9%) (GI); oral administration of distilled water (GII); subcutaneous administration of HA (0.3%) (GIII); and oral administration of HA (1%) (GIV). Flaps of all groups were comparable regarding clinical and macroscopic evaluation, histological examination, pro-inflammatory cytokines (IL-1β, IL-6, and tumor necrosis factor-α) and anti-inflammatory cytokine IL-10.

**Results:**

A lower percentage of necrosis was identified in flaps treated with subcutaneous administration of HA (0.3%). The pro- and anti-inflammatory cytokines, epidermis thickness, blood vessels, and inflammatory cells showed statistically significant inter-group and intra-group differences (*p* < 0.05).

**Conclusions:**

High molecular HA (1,400 ~ 2,000 kDa) administrated by subcutaneous or oral route exhibited beneficial effects in ischemic skin flaps of rats. However, subcutaneous administration of HA (0.3%) showed better results in terms of the percentage of necrosis and epithelialization.

## Introduction

Skin flaps involve the elevation, displacement, and repositioning of the skin in a new wound bed, resulting in a circulatory impairment for a variable and transient period^1^^–^ 3. For flap survival, it is crucial to avoid prolonged ischemia and necrosis, and these factors may be associated with surgical trauma, an exacerbated inflammatory process, and changes resulting from the reestablishment of blood circulation^1^^–^ 5. Consequently, there is a need to use substances that mitigate harmful effects and, in turn, enhance the viability of the skin flaps^1^^–^ 3 ^,^ 5. Several substances have been researched with the aim of minimizing the deleterious effects on skin flaps and improving their viability, and hyaluronic acid has been one of the substances used in this field of research^5^.

Hyaluronic acid (HA) is a biopolymer from glycosaminoglycan and identified in its highest concentration in the skin. It is used in wound healing due to its hydroscopic, homeostatic, immunomodulatory, anti-inflammatory, and antioxidant properties^5^^–^ 15. The mechanism of action of HA is not completely elucidated, but it has already been identified on the the skin and other tissues^16^^–^ 20.

To date, comparisons of exogenous HA administrated by different routes in ischemic flaps are absent in the literature. The aim of this study was to evaluate exogenous high molecular HA (1,400 ~ 2,000 kDa) administrated by subcutaneous or oral route in ischemic skin flaps induced in Wistar rats. The hypothesis is that exogenous HA, whether administered orally or subcutaneously, has beneficial effects on induced ischemic skin flaps.

The findings of the current study could serve as the basis for the use of HA in skin flaps within the field of reconstructive surgery, highlighting the clinical relevance of the study.

## Methods

### Animals and experimental design

This study was approved by the Institutional Ethics Committee for the Use of Animals (CEUA) (Protocol no. 00166/2020).

Sixty-four healthy male Wistar rats, *Rattus norvegicus*, heterogenic, aged between 12 and 14 weeks old, with a mean body mass between 300 and 400 g, were used. The rats were housed at the Experimental Unit of the Department of Morphology of Botucatu Biosciences Institute (Botucatu, São Paulo, Brazil) (GPS: S: 22°53’17,5 WO:48°29’55,4). Additionally, four rats were used as a negative control for the analysis of tissue inflammatory biomarker.

The rats were subjected to acclimatization in the experimental environment for 14 days, in groups of four animals in polysulfone plastic boxes (length = 497 mm, width = 341 mm, height = 265 mm). The animals received a commercial pelleted diet and filtered water *ad libitum*. The environment remained air-conditioned, with temperature control (23ºC), humidity varying between 40 and 60%, and 12-hour light/dark cycles. At this stage, deworming with ivermectin 1% (0.1 mL diluted in 100 mL of drinking water from a collective water fountain), was carried out, every seven days, for a total of 14 days^21^.

After acclimatization, the rats were placed individually in polysulfone boxes (length = 385 mm, width = 251 mm, height = 240 mm), and crumpled bond paper was used as environmental enrichment. The boxes were cleaned every 48 hours with water and neutral soap.

### Anesthesia and surgical procedure of the ischemic skin flaps

**Figure 1:**
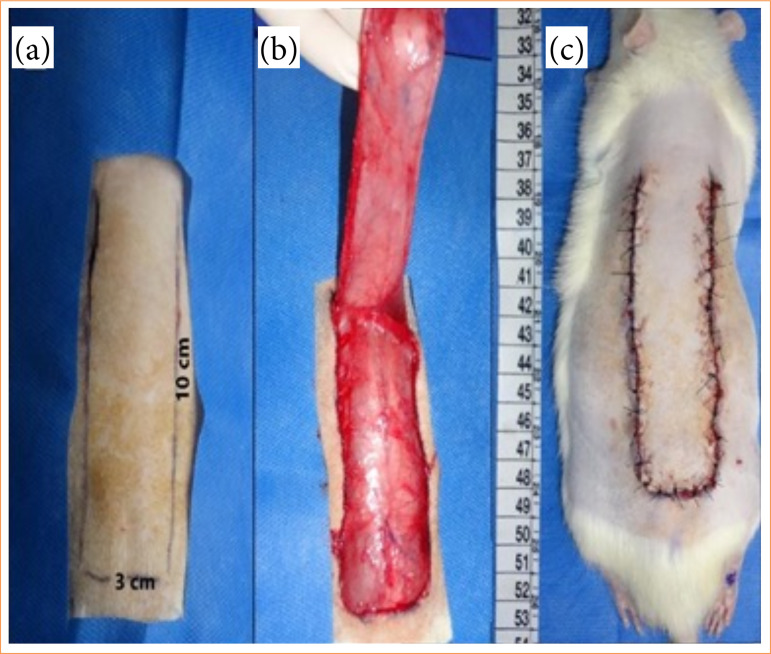
Photography image showing the **(a)** dimension of the skin flap; **(b)** the elevation of the skin flap, and **(c)** the final appearance of the dorsal skin flap after closing with surgical nylon (4-0).

Source: Elaborated by the authors

Anesthesia was performed with a combination of ketamine hydrochloride (75 mg/kg) and xylazine (10 mg/kg) administered intraperitoneally (IP). The rats were placed in ventral recumbence, and, after manual epilation of the dorsal region and aseptic skin preparation of the area with chlorhexidine 2%, a skin incision measuring 3 cm in width and 10 cm in length was made in the region between the scapulae and the base of the tail, using a scalpel (no. 20)^22^ (Fig. 1a). Metzenbaum scissors were employed to elevate the flap (Fig. 1b)^22^, and subsequently, the flaps were closed with a simple interrupted suture using surgical nylon (4-0) (Fig. 1c).

Tramadol hydrochloride [10 mg/kg, each 12 hours (twice a day or each 12 hours), subcutaneous] was administered for 48 hours to manage postoperative pain. The surgical procedures were conducted by the same experienced surgeon at 7 a.m., following the asepsis protocols.

### Treatments

After the skin flaps were created, the rats were randomly assigned to four groups, each consisting of 16 animals, using the Randomizer program (2018), based on the treatment protocol:

- Group I (GI) (control group – subcutaneous route) (G1): 1.5 mL of saline solution 0.9%;
- Group II (GII) (control group – oral route) (G2): 0.9 mL of distilled water;
- Group III (GIII) (experimental group – subcutaneous route) (G3): 1.5 mL of HA 0.3%;
- Group IV (GIV) (experimental group – oral route) (G4): HA 1% (0.025 mg/g).

The HA (1,400 ~ 2,000 kDa) (lyophilized), derived from bacterial fermentation with an injectable degree of purity, was used. The dilution was performed 5 minutes before the use. For oral and subcutaneous administration, dilutions to 1 and 0.3% were made with sterile distilled water, respectively.

A single dose of saline solution 0.9% (G_I_) and HA 0.3% (G_III_) was administered under the flaps after their closure. For this purpose, an atraumatic microcannula (0.90 mm × 100 mm) was used and inserted between the sutures in a craniocaudal direction.

Distilled water (G_II_) and HA 1% (G_IV_) were administered through gavage using a curved cannula (1.20 mm × 39 mm), starting eight hours before the skin flaps were created, and repeated every 24 hours for seven days^18^^,^ 19. All treatments were administered at 7 a.m., and the study was conducted as a double-blinded study. No antibiotics or anti-inflammatories were administered during the study.

### Clinical evaluation

The clinical evaluation included the body mass (BM) and rectal temperature (RT) measurement; macroscopic evaluation of the skin flaps (suture dehiscence and seroma); and percentage of necrosis. BM and RT were measured using a precision digital scale and a digital rectal thermometer, respectively. Suture dehiscence and seroma were assessed through clinical observation and classified based on the presence or absence of these alterations.

The percentage of necrosis was evaluated using the software ImageJ. For this purpose, the flaps were photographed with a digital camera (16.2-megapixel resolution) in JPEG format. The camera was positioned perpendicularly to the skin flaps using a specific support, maintaining a standardized height of 25 cm between the camera and the flaps. Simultaneously, a measuring tape (100-cm long) was placed on the animal’s side to serve as a metric reference for calculating the areas. Three photographs were taken to obtain an average value.

The percentage of necrosis was determined using Eq. 122:


$$\text{Percentage of necrosis}=\frac{\text{Necrotic area}}{\text{Total flap area}}\times 100\%$$ (1)

The BM, RT, and percentage of necrosis were evaluated in the following time points: 10 minutes before the creation of the skin flaps (M_0_), three days (M_3d_) and seven days (M_7d_) after the creation of the skin flaps. The presence of suture dehiscence and seroma was determined every 24 hours for seven days.

### Inflammatory cytokines and histological evaluation

Eight rats from each group were randomly selected and euthanized using a combination of xylazine 2% (30 mg/kg) and ketamine hydrochloride 1% (180 mg/kg) (IP) at the following time points: M_3d_ and M_7d_. Flap specimens for inflammatory cytokines and histological evaluation were collected from the cranial region of the necrotic area. The pro-inflammatory cytokines evaluated were interleukin (IL)-1β, IL-6, and tumor necrosis factor (TNF)-α, and the IL-10 was used as an anti-inflammatory cytokine.

The samples for evaluating pro- and anti-inflammatory cytokines were placed in Eppendorf tubes, immersed in liquid nitrogen (-196°C), and stored at -80°C. The samples were homogenized in a 1:5 ratio with phosphate-buffered saline (pH = 7.4) and a protease inhibitor cocktail (99:1) (Sigma-Aldrich). Subsequently, the homogenate was centrifuged for 15 minutes at 10,000 rpm and 4°C. After centrifugation, the supernatant liquid was collected for protein quantification and analysis. Total protein quantification was performed using the protein detection kit (Interteck Katal) through the biuret method, following the company’s instructions. Cytokine levels were determined using enzyme-linked immunosorbent assay (ELISA) with commercial kits for each cytokine (R&D Systems), following the protocols and concentrations provided by the company.

The specimens for histological evaluation were immersed in an Alfac solution for 24 hours and subsequently preserved in 70% alcohol until embedded in paraffin. Sections of 5 µm were prepared and stained using the hematoxylin-eosin (H&E) method for general tissue evaluation. The quantity of total collagen was determined through Masson’s Trichrome staining.

For histological evaluation, the analyses were conducted using photomicrographs of the central region of the flaps, capturing 10 fields at 40x magnification, totaling an area of 100,000 µm^2^. The morphometric analysis included the count of blood vessels/field, total inflammatory cells/field, and total collagen/field. For the epidermal thickness (µm), 10 measurements/field were taken. Morphometric assessments were performed using cellSens Standard software, and measurements were taken using AVSoft BioView software.

### Statistical analysis

For statistical analysis, R (Version 4.0.5, 2021) and Excel (Version 16.0.6742.2048, 2019) softwares were used. Descriptive statistics for quantitative variables included mean, standard deviation, median, maximum and minimum values, as well as first and third quartiles. The normality of results was assessed using the Shapiro-Wilk’s test. In cases in which the assumption of normality was present, the parametric T-test for dependent and independent samples was used. In cases in which the assumption of normality was not present, the Wilcoxon’s test was applied. The analysis of variance test was used for group comparisons when the assumption of normality was present, and the Kruskal-Wallis’ test was used when normality was not assumed. A significance level of less than 0.05 was considered for all comparisons.

## Results

### Clinical assessment

During the period of the study, no decrease in food and water intake was identified, nor the presence of diarrhea or deaths. At the same time, no skin reactions were observed resulting from the application of HA.

The BM did not demonstrate significant variation between the groups. However, there was a significant decrease (*p* = 0.0001) in all groups between M0 and M3d (Table 1). RT values showed no statistical differences (Table 2).

**Table 1:** Median (Med) and values [minimum (min) and maximum (max)] of body mass **(g)** of Wistar rats with ischemic skin flaps and treated with saline solution (0.9%) subcutaneous (GI), distilled water orally (GII), hyaluronic acid (0.3%) subcutaneous (GIII), and hyaluronic acid (1%) orally (GIV), and evaluated 10 minutes before creating the skin flaps (M0), third day (M3d) and seventh day (M7d) after the creation of the flaps*.

Groups	M0 Med (min–max)	M3d Med (min–max)	M7d Med (min–max)
GI	342.2 (301.6–370.7)^Aa^	333.7 (288.1–350.9)^Ba^	337.5 (323.5–383.4)^ABa^
GII	346.3 (331.3–370.8)^Aa^	334.5 (306.2–362.3)^Ba^	346.9 (331.4–392.1)^ABa^
GIII	341.0 (303.5–370.5)^Aa^	329.6 (291.5–365.9)^Ba^	347.2 (307.9–372.7)^ABa^
GIV	340.9 (284.1–399.7)^Aa^	324.4 (280.4–390.7)^Ba^	343.3 (329.6–407.1)^ABa^

* Medians followed by different capital letters in the same line represent a significant difference across treatments (*p* < 0.05).

Medians followed by different lowercase letters in the same column represent differences between groups (*p* < 0.05). Source: Elaborated by the authors.

**Table 2:** Median (Med) and values [minimum (min) and maximum (max)] of rectal temperature values (°C) of Wistar rats with ischemic skin flaps and treated with saline solution (0.9%) subcutaneous (GI), distilled water orally (GII), hyaluronic acid (0.3%) subcutaneous (GIII), and hyaluronic acid (1%) orally (GIV), and evaluated 10 minutes before creating the skin flaps (M0), third day (M3d) and seventh day (M7d) after the creation of the flaps.

Groups	M3d Med (min–max)	M7d Med (min–max)
GI	13.58 (3.78–36.04)^Aa^	33.80 (21.19–56.19)^Bab^
GII	17.20 (0.70–34.01)^Aa^	42.13 (16.41–56.56)^Bab^
GIII	10.52 (1.76–26.46)^Aa^	26.325 (8.55–51.05)^Ba^
GIV	18.56 (0.00–38.70)^Aa^	50.401 (12.61–77.80)^Bb^

* Medians followed by different capital letters in the same line represent a significant difference across treatments (*p* < 0.05).

Medians followed by different lowercase letters in the same column represent differences between groups (*p* < 0.05). Reference values: 35.9–37.5°C. Source: Elaborated by the authors.

There was no evidence of seroma in any of the groups. In the other hand, dehiscence of a suture was identified in the distal region to the base of the flap, 24 hours later, however, without resulting in flap opening. The dehiscence in group treated with HA (1%) by orally route was of 62.5% (10/16); 56.3% (9/16) in the group treated with HA (0.3%) by subcutaneous route; 50% (8/16) in the group treated with distilled water orally; and 37.5% (6/16) in the group treated with saline solution (0.9%) by subcutaneous route.

**Figure 2:**
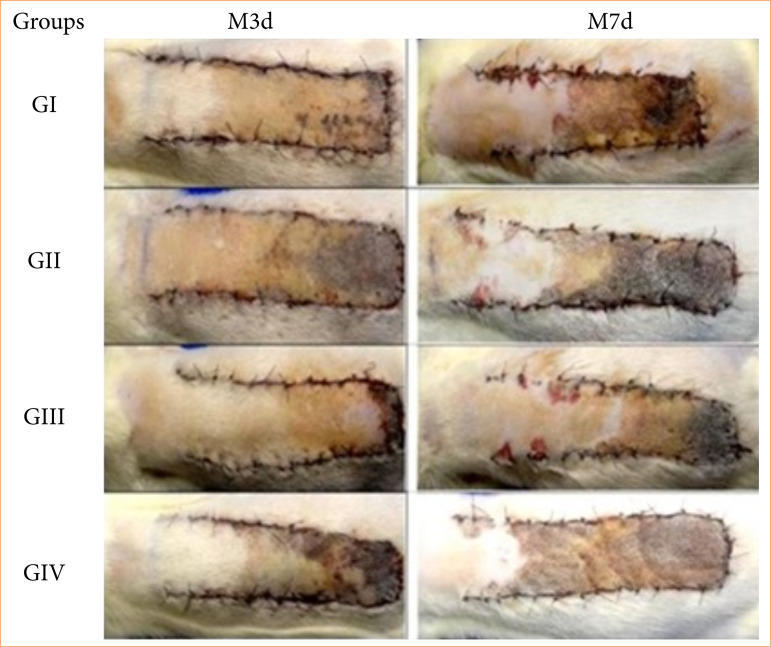
Macroscopic aspect of necrosis of ischemic dorsal skin flaps treated with saline solution (0.9%) subcutaneous (GI), distilled water orally (GII), hyaluronic acid (0.3%) subcutaneous (GIII) and hyaluronic acid (1%) orally (GIV) and evaluated on the third day (M3d) and on the seventh day (M7d) after the creation of the flaps.

Source: Elaborated by the authors.

In all animals, the onset of necrosis occurred 48 hours after flap creation, with an increase in the necrotic area up to seven days after flap induction (Fig. 2). All groups showed a statistical difference (*p* = 0.0019) regarding the percentage of necrosis, with an increase being identified between M3d and M7d. In the evaluation between groups, a significant difference was identifiy between the animals in the group subjected to treatment with HA (0.3%) subcutaneous (G_III_) and the groups treated with HA (1%) orally (G_IV_) (G_IV_ > G_III_) (*p* = 0.00011), seven days after flap induction (Table 3).

**Table 3:** Median (Med) and values [minimum (min) and maximum (max)] of the percentage of necrosis (%) of Wistar rats with ischemic skin flaps and treated with saline solution (0.9%) subcutaneuos (GI), distilled water orally (GII), hyaluronic acid (0.3%) subcutaneous (GIII), and hyaluronic acid (1%) orally (GIV), and evaluated 10 minutes before creating the skin flaps (M0), third day (M3d) and seventh day (M7d) after the creation of the flaps*.

Groups	M3d Med (min–max)	M7d Med (min–max)
GI	13.58 (3.78–36.04)^Aa^	33.80 (21.19–56.19)^Bab^
GII	17.20 (0.70–34.01)^Aa^	42.13 (16.41–56.56)^Bab^
GIII	10.52 (1.76–26.46)^Aa^	26.325 (8.55–51.05)^Ba^
GIV	18.56 (0.00–38.70)^Aa^	50.401 (12.61–77.80)^Bb^

* Medians followed by different capital letters in the same line represent a significant difference across treatments (*p* < 0.05).

Medians followed by different lowercase letters in the same column represent differences between groups (*p* < 0.05). Source: Elaborated by the authors.

Assessment of inflammatory tissue biomarkers (inflammatory cytokines)

The IL-1β values demonstrated a significant increase in flaps treated with saline solution (0.9%) subcutaneous (G_I_) between both time points. The evaluation between groups identified significant variations at both time points: M_3d_ - G_I_, G_III_ and G_IV_ > G_II_; M_7d_ – G_I_ > G_II_ (*p* = 0.0001) (Table 4).

**Table 4:** Median (Med) and values [minimum (min) and maximum (max)] of IL-1β (pg/mg protein) and IL-6 (pg/mg protein) and TNF-α (pg/mg protein) of ischemic skin flaps treated with saline solution (0.9%) subcutaneous (GI), distilled water orally (GII), hyaluronic acid (0.3%) subcutaneous (GIII) and hyaluronic acid (1%) orally (GIV) and evaluated on the third day (M3d) and on the seventh day (M7d) after the creation of the flaps*.

Pro-inflammatory interleukins	Groups	M3d Med (min–max)	M7d Med (min–max)
IL-1β	GI	41.88 (34.69–55.86)^Aa^	64.70 (50.22–79.66)^Ba^
	GII	17.96 (7.01–27.53)^Ab^	22.60 (16.07–30.43)^Ab^
	GIII	39.14 (24.44–64.43)^Aa^	45.45 (15.81–91.38)^Aab^
	GIV	44.88 (28.54–65.65)^Aa^	45.39 (42.72–66.782)^Aab^
IL-6	GI	59.59 (52.48–67.52)^Aa^	83.42 (56.41–189.95)^Ba^
	GII	26.33 (13.48–37.14)^Ab^	35.85 (23.74–40.58)^Bc^
	GIII	52.22 (31.76–98.74)^Aa^	64.02 (36.37–127.36)^Aa^
	GIV	59.99 (25.74–108.03)^Aa^	66.19 (34.23–98.31)^Aabc^
TNF-α	GI	12.35 (5.18–19.43)^Aa^	17.21 (14.12–37.31)^Ba^
	GII	4.64 (2.24–7.89)^Ac^	6.05 (4.02–9.65)^Ab^
	GIII	9.37 (4.14–28.17)^Aabc^	14.35 (5.59–34.44)^Aab^
	GIV	10.50 (8.22–28.59)^Aab^	11.40 (2.64–30.57)^Aab^

IL: interleukin; TNF: tumor necrosis factor;

* medians followed by different capital letters on the same line represent a significant difference across treatments (*p* < 0.05).

Medians followed by different lowercase letters in the same column represent differences between groups (*p* < 0.05). Laboratory reference: IL-1β = 10.68–18.32 pg/mg protein; IL-6 = 19.44–38.64 pg/mg of protein; TNF-α = 2.38–9.06 pg/mg of protein. Source: Elaborated by the authors.

Regarding IL-6 values, a significant increase (*p* = 0.00014) was identified in flaps treated with parenteral saline solution (0.9%) (G_I_) and in those treated with distilled water enterally (G_II_) between the time points; however, the values of the G_II_ remained within the species’ reference range. At the same time, in M3d significant variations (*p* = 0.00011) were evident similar to those observed in the assessment of IL-1β. In M_7d_, the following statistical variations were identified: G_I_ and G_III_ > G_II_ (*p* = 0.00011) (Table 4).

The TNF-α values showed significant variations (*p* = 0.0001) between both time points, demonstrating a significant increase in the group treated with saline solution (0.9%) subcutaneous (GI). In the evaluation between groups, significant variations (*p* = 0.0002) were found in the all-time points evaluated: M_3d_ - G_I_ and G_IV_ > G_II_; M_7d_ - G_I_ > G_II_ (Table 4).

In tissue quantification of the anti-inflammatory biomarker (IL-10), statistical variations (*p* = 0.0001) were identified between the group treated with saline solution (0.9%) subcutaneous (G_I_) and the group treated with distilled water orally (G_II_) at both time points, with the values of the G_I_ being significantly higher than G_II_ (Table 5).

**Table 5:** Median (Med) and values [minimum (min) and maximum (max)] of the IL-10 value (pg/mg protein) of ischemic skin flaps treated with saline solution (0.9%) subcutaneous (GI), distilled water orally (GII), hyaluronic acid (0.3%) subcutaneous (GIII) and hyaluronic acid (1%) orally (GIV) and evaluated on the third day (M3d) and on the seventh day (M7d) after the creation of the flaps*.

Groups	M3d Med (min–max)	M7d Med (min–max)
GI	13.57 (0.00–20.90)^Aa^	16.02 (11.42–28.29)^Aa^
GII	5.05 (0.00–7.92)^Ab^	5.91 (3.44–8.29)^Ab^
GIII	11.37 (0.00–21.49)^Aab^	14.10 (0.00–27.99)^Aab^
GIV	11.87 (0.00–24.85)^Aab^	12.81 (0.00–22.14)^Aab^

* Medians followed by different capital letters on the same line represent a significant difference across treatments (*p* < 0.05).

Medians followed by different lowercase letters in the same column represent differences between groups (*p* < 0.05). Laboratory reference: IL-10 = 0.00–7.83 pg/mg protein. Source: Elaborated by the authors.

### Histological evaluation

Epidermal thickness values demonstrated a significant increase (*p* = 0.0001) in flaps treated with saline solution (0.9%) subcutaneous (G_I_) and with HA (1%) orally (G_IV_) between the time points. In the inter-group evaluation, it was observed that G_III_ presented significantly higher thickness values (*p* = 0.004) than the other groups in M_3d_; and in M_7d_. Significantly higher values (*p* = 0.0041) were identified in the G_III_ compared to the G_II_ (Table 6).

**Table 6:** Median (Med) and values [minimum (min) and maximum (max)] of epidermis thickness (μm) number of blood vessels (n/field), number of total inflammatory cells (μm2) and total collagen (μm^2^) of Wistar rats with ischemic skin flaps and treated with saline solution (0.9%) subcutaneous (GI), distilled water orally (GII), hyaluronic acid (0.3%) subcutaneous (GIII) and hyaluronic acid (1%) orally (GIV) and evaluated on the third day (M3d) and on the seventh day (M7d) after the creation of the flaps*.

Histological variables	Groups	M3d Med (min–max)	M7d Med (min–max)
Epidermis thickness	GI	25.18 (12.57–49.84)^Aa^	28.975 (15.06–68.61)^Bab^
Epidermis thickness	GII	25.73 (13.82–61.11)^Aa^	26.78 (12.95–65.65)^Ab^
Epidermis thickness	GIII	28.23 (13.81–52.98)^Ab^	29.39 (15.09–61.98)^Aa^
Epidermis thickness	GIV	25.83 (10.45–50.71)^Aa^	27.74 (11.79–84.46)^Bab^
Blood vessels	GI	5.7 (4.6–7.0)^Aab^	4.6 (4–6.6)^Ba^
Blood vessels	GII	4.8 (3.8–5.7)^Aa^	4.4 (3–4.9)^Aa^
Blood vessels	GIII	4.4 (3.2–7.5)^Aab^	4.7 (3.2–6.3)^Aa^
Blood vessels	GIV	6.8 (4.5–12)^Ab^	4.4 (3.7–9.4)^Ba^
Total inflammatory cells	GI	2,750.4 (1,347.0–3,293.1)^Aa^	2,721.8 (1,997.4–4,406.2)^Aa^
Total inflammatory cells	GII	1,839.7 (1,603.5–2,321.8)^Aa^	1,828.4 (1,572.9–2,597.5)^Ab^
Total inflammatory cells	GIII	2,284.9 (1,155.4–3,294.5)^Aa^	2,837.6 (1,615.9–3,412.3)^Aab^
Total inflammatory cells	GIV	2,742.8 (1,435.2–3,232.7)^Aa^	2,200.4 (1,984.8–3,840.2)^Aab^
Total collagen	GI	31,506.4 (23,279.2–40,073.0)^Aa^	26,956.0 (20,629.0–32,246.2)^Aa^
Total collagen	GII	24,026.2 (19,865.6–38,274.8)^Aa^	24,667.6 (17,430.8–29,823.5)^Aa^
Total collagen	GIII	31,070.1 (26,136.6–34,087.6)^Aa^	25,497.1 (24,098.2–37,653.1)^Aa^
Total collagen	GIV	30,232.3 (22,819.7–42,584.9)^Aa^	28,336.3 (25,276.4–33,620.6)^Aa^

* Medians followed by different capital letters on the same line represent a significant difference across treatments (*p* < 0.05).

Medians followed by different lowercase letters in the same column represent differences between groups (*p* < 0.05). Source: elaborated by the authors.

Regarding the quantification of blood vessels, there was a significant decrease (*p* = 0.004) in flaps treated with saline solution (0.9%) parenterally (G_I_) and with HA (1%) enterally (GIV) between the time points. Only at M_3d_ a statistical variation (*p* = 0.0041) was identified where the group treated with HA (1%) enterally (G_IV_) presented higher values than the group treated with distilled water orally (G_II_) (Table 6).

Concerning the number of total inflammatory cells, significant variations (*p* = 0.0045) were identified at M_7d_ with G_I_ > G_II_ (Table 6).

**Figure 3:**
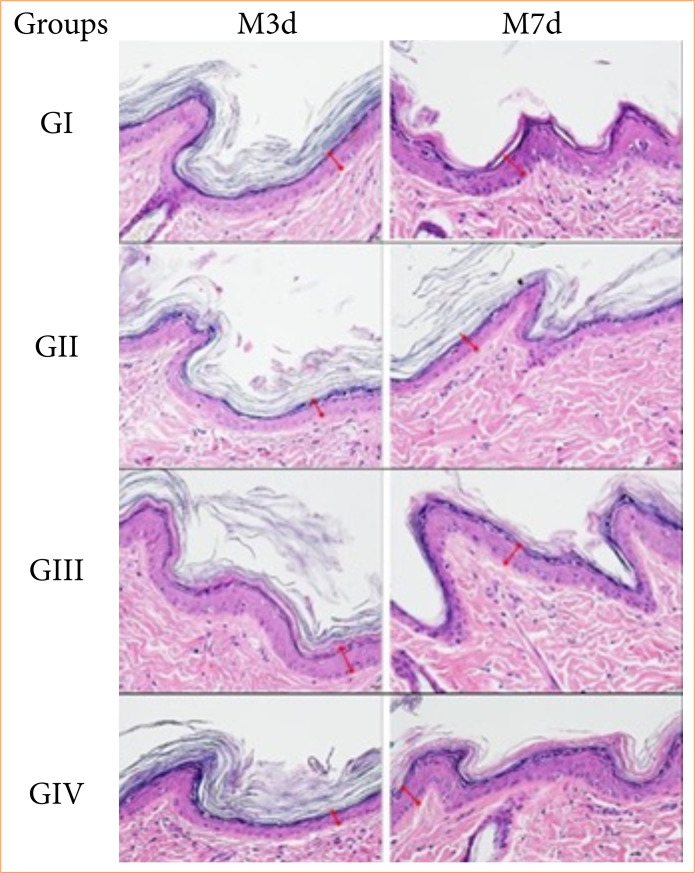
Photomicrographs of measuring the thickness of the epidermis (red demarcation) of ischemic flaps treated with saline solution (0.9%) subcutaneous (GI), distilled water orally (GII), hyaluronic acid (0.3%) subcutaneous (GIII) and hyaluronic acid (1%) orally (GIV) and evaluated on the third day (M3d) and on the seventh day (M7d) after the creation of the flaps (hematoxylin and eosin. 40x, scale bar = 20 µm).

Source: Elaborated by the authors.

**Figure 4:**
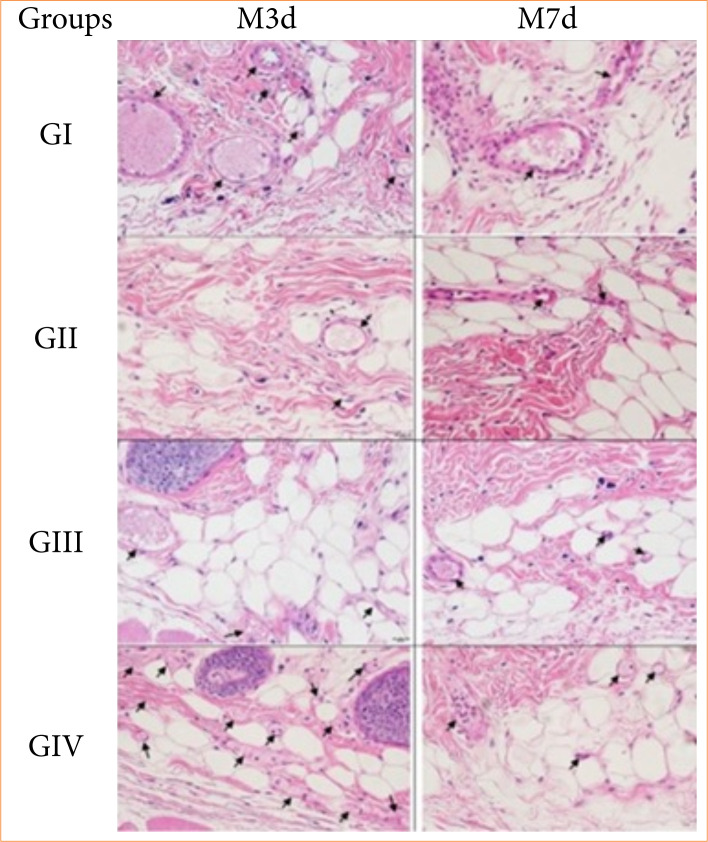
Photomicrographs of the deep dermis region of ischemic flaps treated with saline solution (0.9%) subcutaneous (GI), distilled water orally (GII), hyaluronic acid (0.3%) subcutaneous (GIII) and hyaluronic acid (1%) orally (GIV) and evaluated on the third day (M3d) and on the seventh day (M7d) after the creation of the flaps. Illustrating blood vessels (black arrows) and inflammatory cells (purple-stained nuclei) (hematoxylin and eosin, 40x, scale bar = 20 μm).

Source: Elaborated by the authors.

**Figure 5:**
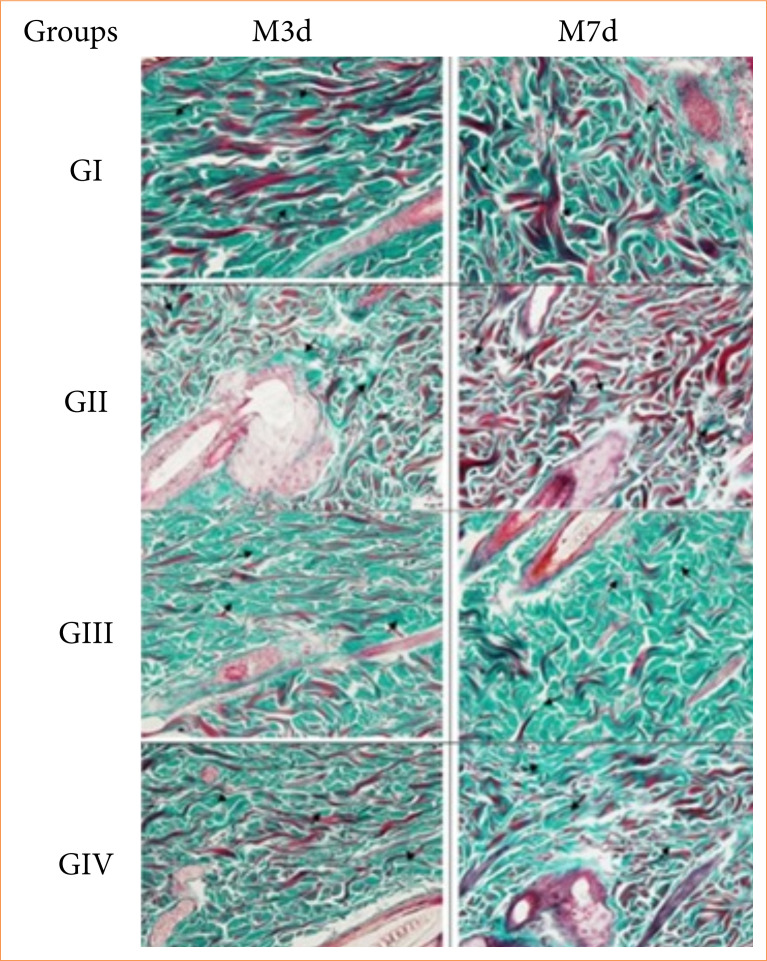
Photomicrographs of the dermal region of ischemic flaps treated with saline solution (0.9%) subcutaneous (GI), distilled water orally (GII), hyaluronic acid (0.3%) subcutaneous (GIII) and hyaluronic acid (1%) orally (GIV) and evaluated on the third day (M3d) and on the seventh day (M7d) after the creation of the flaps, illustrating the collagen fibers stained in green (Masson’s tricrome, 40x, scale bar = 20 μm).

Source: Elaborated by the authors.

The analysis of total collagen presented no significant intragroup and intergroup differences (Table 6). Figures 3, 4 and 5 represent photomicrographs of the histological variables evaluated in all groups at both time points.

## Discussion

The aim of the study was to evaluate high molecular HA (1,400 ~ 2,000 kDa) derived from bacterial fermentation by enteral and parenteral routes in ischemic skin flaps in rats, through clinical and histological examinations and by evaluation of tissue inflammatory biomarkers. The hypothesis was partially achieved, since, according to the results, the group which HA (0.3%) was administered locally showed better beneficial effects regarding the percentage of necrosis when compared to the other groups. The use of substances with immunomodulatory or anti-inflammatory properties in skin flaps aims to modulate exacerbated inflammation resulting from ischemic and/or oxidative events and thus to avoid tissue damage and subsequent necrosis^1^^–^ 3 ^,^ 23 ^,^ 24.

The study was considered unprecedented since the evaluation of exogenous HA in ischemic skin flaps, whether enterally or parenterally routes, is absent in the literature. However, studies related to the use of HA in open wounds and corneal ulcers were reported by different authors^7^^,^ 10 ^,^ 12 ^,^ 25 ^–^ 28. The clinical relevance of the present study was associated with the anti-inflammatory, antioxidant properties, and the stimulation of neovasculogenesis of HA^8^^,^ 13 ^,^ 14 ^,^ 29, which may induce beneficial effects on the viability of the skin flaps.

The short-term experimental model was similar to the literature^1^^,^ 3 ^,^ 30 ^–^ 33, and the methodology for creating ischemic flaps was carried out according to the model proposed by Camargo et al.^22^.

The concentration of exogenous HA (0.3%) used by subcutaneous route was according to the literature, by *in-vitro* and *in-vivo* studies^34^^,^ 35. Finally, the oral HA concentration (1%) was determined by the dose and volume of the gastric capacity of rats^36^.

The enteral administration of HA was carried out eight hours before creating the flap, because its presence in the skin of rats was identified four hours after its administration^16^, and its peak bioavailability was observed after eight hours^19^.

The RT remained within the species reference limits in all groups throughout the study period. This fact may have indicated the absence of contamination or infection in the flaps^37^. Even though microbiological tests were not performed, the absence of contamination was correlated with the macroscopic evaluation of the flaps, and no clinical signs associated with contamination were observed. However, a significant decrease in BM was observed in all groups, between the day of flap creation and three days after flap induction. This decrease was possibly due to catabolism associated with the inflammatory response and the stress of the surgical procedure^38^.

Suture dehiscence was identified in all groups after 24 hours of the flap induction, with a higher incidence in flaps treated with HA, and it was associated with self-trauma observed during the study. However, these dehiscences did not influence with the fixation of the flaps. According to Huang et al.^1^ and Feng et al.^3^, the wide extension of the flaps on the back of the rats makes access to the suture stitches possible, with it being considered a negative point in studies with dorsal skin flaps. The same authors^1^^,^ 3 used a cervical collar to prevent the rats from reaching the sutures, however, in the present study, it was decided not to use such device, as it could prevent self-cleaning and induce stress in the animals^37^^,^ 39.

The necrosis was identified 48 hours after flap induction, and the significant increase was observed following seven days of evaluation, and was correlated with possible persistent ischemia due to subdermal vascular compromise^1^^,^ 3 ^,^ 30 ^–^ 33 ^,^ 39 ^–^ 42. On the other hand, the higher percentage of necrosis in rats treated with HA (1%) by oral route was associated with the possible bacterial intestinal fragmentation of the substance that may have occurred in the animals, determining a lower concentration of it in the flaps^35^. Balogh et al.^16^ identified the presence of radioactive HA in the skin of rats after four hours of oral administration of high molecular weight HA (1,100 – 1,500 kDa), and this fact was corroborated by Oe et al.^17^ and Kimura et al.^19^.

All groups demonstrated an increase in the values of pro-inflammatory cytokines (IL-1β, IL-6 and TFN-α), throughout the evaluated time points, and was associated with vascular reperfusion injury that occurs in ischemic flaps^1^^,^ 3 ^,^ 23, and surgical trauma, as an inflammatory response^38^^,^ 44, however, a balanced production is necessary^43^^–^ 45.

The variation in the values of the anti-inflammatory interleukin (IL-10) demonstrated a possible limitation of the exacerbated inflammatory response associated with IL-1β, IL-6 and TFN-α^44^^–^ 48.

The epidermis is the skin layer that is most affected by ischemic events in skin flaps, which can cause epitheliolysis and partial loss of the flap^22^^,^ 39 ^,^ 40. Only the flaps treated with saline solution (0.9%) and enteral HA showed a significant increase in epidermis thickness over time. However, rats treated with HA by subcutaneous route showed significantly greater values of epidermis thickness when compared with the other groups. These results were associated with the increased stimulation of keratinization and epithelialization guaranteed by HA^29^.

The total inflammatory cells in all groups, except rats treated with parenteral HA, followed the same pattern as pro-inflammatory cytokines. Iacopetti et al.^12^ showed that the topical use of HA in wounds stimulated more hair follicles, which could negatively interfere with the measurement of cellularity of flaps treated with HA parenterally.

The decrease of blood vessels in histological quantification was similar to the literature^1^^,^ 3 ^,^ 30 ^–^ 33 ^,^ 39 ^,^ 40. These authors associated this fact with subdermal vascular damage. It was highlighted that the flaps treated with HA by oral route presented a significantly higher value compared to those submitted to distilled water orally. These values were related to the increase in pro-inflammatory cytokines, which could determine the bioavailability of HA in the skin^17^^–^ 19.

No significant differences were identified between the groups and time points evaluated regarding to total collagen. However, exogenous HA increased collagen production when used in dermal fibroblast cultures^42^, and intradermal application^47^^,^ 48. This increase was due to the compressive stimulus of HA on the fibroblasts^48^.

The limitation of the study included the absence of an immunohistochemical exams to measure the endothelial growth factor, which could allow the quantification of blood vessels objectively. Therefore, there is the need to include this exam in similar studies.

## Conclusion

The high molecular weight (1,400 ~ 2,000 kDa) HA demonstrates beneficial effects when used by oral and subcutaneous route in ischemic skin flaps from rats. However, HA (0.3%) administered subcutaneous route at the flap site shortly after its closure shows better results in the percentage of necrosis and the epithelialization process.

## Data Availability

All data were generated or analyzed in the current study.
